# The analgesic effects of Yu-Xue-Bi tablet (YXB) on mice with inflammatory pain by regulating LXA4-FPR2-TRPA1 pathway

**DOI:** 10.1186/s13020-024-00975-1

**Published:** 2024-08-06

**Authors:** Ying Liu, Guoxin Zhang, Chunyan Zhu, Xuemin Yao, Wenli Wang, Li Shen, Haiping Wang, Na Lin

**Affiliations:** 1 Institute of Chinese Materia Medica, China Academy of Chinese Medical Sciences, Beijing, 100700 China; 2 Institute of Basic Theory for Chinese Medicine, China Academy of Chinese Medical Sciences, Beijing, 100700 China

**Keywords:** Yu-Xue-Bi tablet, Inflammatory pain, Oxylipins, Lipoxins A4, Inflammation resolution

## Abstract

**Background:**

Oxylipins including lipoxin A4 (LXA4) facilitate the resolution of inflammation and possess analgesic properties by inhibiting macrophage infiltration and transient receptor potential (TRP) protein expression. Yu-Xue-Bi Tablet (YXB) is a traditional Chinese patent medicine used to relieve inflammatory pain. Our previous research has shown that the analgesic effect of YXB is related to inhibiting peripheral inflammation and regulating macrophage infiltration, but the mechanism is not yet clear. The purpose of this study is to explore the mechanisms of YXB on mice models with Complete Freund’s Adjuvant (CFA)-induced inflammatory pain from the perspective at the resolution of inflammation.

**Methods:**

Mechanical allodynia thresholds and heat hypersensitivity were measured using the Von Frey test and the hot plate test respectively. The open field test and the tail suspension test were employed to measure anxiety and depressive behaviors respectively. The expression of CD68^+^ and the proportion of F4/80^+^CD11b^+^ cells were measured by immunofluorescence staining and flow cytometry. The expression of transient receptor potential ankyrin 1(TRPA1) was measured by immunofluorescence staining and western blotting. Oxylipins omics analysis provided quantitative data on oxylipins in the paws, and enzyme linked immunosorbent assay (ELISA) was used to measure the levels of LXA4 there. Immunofluorescence staining was used to perform the expression of Leukotriene A4 hydroxylase (LTA4H) in the paws of mice. The impact of injecting the formyl peptide receptor 2(FPR2) antagonist WRW4 and the TRPA1 agonist AITC into the left paws was observed, focusing on the expression of mechanical allodynia thresholds, the expression of CD68^+^, TRPA1 in the paws, and Calcitonin gene-related peptide (CGRP) in the L5 spinal dorsal horn.

**Results:**

YXB elevated mechanical allodynia thresholds, alleviated heat hypersensitivity and anxiety and depressive behaviors in CFA mice. It significantly reduced the number of CD68^+^ and proportion of F4/80^+^CD11b^+^ within the paws, thereby decreasing macrophage infiltration. Additionally, it diminished the expression of TRPA1 in the paws and TRPV1 in the DRG, leading to an inhibition of peripheral sensitization. Through quantitative analysis, it was found that YXB could modulate DHA-derived oxylipins and LXA4. ELISA results indicated that YXB elevated the levels of LXA4 and inhibited the expression of LAT4H in the paws. Furthermore, the pro-resolution and analgesic effects of YXB were hindered after administration of the FPR2 antagonist. Compared with the AITC group, YXB showed no significant improvement in anti-inflammatory and analgesic effects.

**Conclusions:**

YXB can regulate the oxylipins of paws in CFA mice to promote the resolution of inflammation. The LXA4-FPR2-TRPA1 pathway is a key mechanism for the resolution of inflammation and analgesic effects.

**Supplementary Information:**

The online version contains supplementary material available at 10.1186/s13020-024-00975-1.

## Introduction

Inflammatory pain refers to increased sensitivity caused by inflammation and immune response associated with tissue damage, and it can be found in a variety of conditions such as rheumatoid arthritis (RA), osteoarthritis (OA) and myofascial pain syndrome [1–3]. The prolonged and persistent pain severely impacts the health and quality of life of patients, leading to negative psychological state such as anxiety and depression [4]. Ibuprofen (IBU), a representative of non-steroidal anti-inflammatory drugs (NSAIDs), is the primary approach to treating inflammatory pain. However, IBU is restricted to the treatment of acute inflammatory pain, and its effect on chronic inflammatory pain is limited [5]. Moreover, it may even increase the risk of transitioning inflammatory pain from the acute phase to the chronic phase [6]. In addition, long-term use may also give rise to adverse reactions such as gastrointestinal bleeding and cardiovascular risks [7, 8]. Therefore, it is necessary to seek out an active and effective treatment for inflammatory pain, especially in the chronic phase.

Studies in mounting numbers have indicated that the timely resolution of inflammatory response is a positive and highly coordinated process [9]. Specific endogenous mediators are involved in regulating the inflammation resolution, which not only terminates the inflammatory response autonomously, but also promotes tissue repair and providing analgesic effects [10]. The absence of effective and timely response will lead to the transformation of inflammation from acute into chronic phase [11]. Persistent chronic inflammation is a crucial reason for patients to endure long-term severe pain, therefore, promoting inflammation resolution is becoming an important treatment strategy for inflammatory pain [12].

During the inflammation resolution, oxylipins act as endogenous signaling molecules.

Oxylipins, a diverse group of oxidative metabolites, are synthesized through the auto-oxidation of polyunsaturated fatty acids or by enzymatic processes involving cyclooxygenase (COX), lipoxygenase (LOX), and cytochrome P450 (CYP450) enzymes. These processes also yield specialized pro-resolving mediators (SPMs) such as resolvins (Rv), maresins (Mar), lipoxins (LX), and protectins [13]. Several researches have showed that oxylipins can promote inflammation resolution and alleviate pain directly or indirectly [14, 15]. On the one hand, SPMs such as LXA4, RvD1, Mar1 and RvD2 have been demonstrated to play a role in pro-resolving and relieving pain in both the peripheral and central nervous systems [16–18]. On the other hand, oxylipins can modulate the function of macrophages, microglia, and neurons by activating their respective receptors, and thus promoting inflammation resolution. Further, the signal transduction of oxylipin receptors in the cell body, peripheral and central terminals of nociceptors can reduce the activity of transient receptor potential ankyrin 1 (TRPA1) and transient receptor potential vanilloid subfamily 1 (TRPV1), which contributes to analgesic effects [19].

Traditional Chinese medicine (TCM), with its unique characteristics, is a primary component of the world’s traditional medicine that plays a significant role in the treatment of inflammatory pain. In TCM terminology, inflammatory pain is defined as "Bi syndrome". Yu-Xue-Bi Tablet (YXB) is a prescription medication for treating Bi syndrome in clinical practice, and has been approved by the China Food and Drug Administration (YBZ28152005). It is composed of 11 Chinese herbal medicines, namely Olibanum (Boswellia carteri Birdw.), myrrha (Commiphora myrrha (Nees) Eng), Salviae miltiorrhizae radix et rhizome (Salvia miltiorrhiza Bunge), Clematidis radix et rhizome (Clematis chinensis Osbeck), Chuanxiong rhizome (Ligusticum chuanxiong Hort), Carthami flos(Carthamus tinctorius L.), Angelicae sinensis radix (Angelica sinensis (Oliv.) Diels), Cyathulae radix (Cyathula offfcinalis Kuan), Curcumae longae rhizome (Curcuma longa L.), Cyperi rhizome (Cyperus rotundus L.) and Astragali radix (Astragalus membranaceus (Fisch.) Bunge). According to clinical investigation, YXB has positive effects on OA and RA, which can not only effectively relieve joint pain, but reduce the expression of patients’ serum C-reactive protein, interleukin-8 (IL-8), IL-6, IL-1, tumor necrosis factor-α(TNF-α), intercellular cell adhesion molecule-1(ICAM-1), and facilitate foster inflammation resolution[20, 21].In terms of animal experimental, it has been proven that YXB can intervene in RA by inhibiting angiogenesis through suppressing LOX/Ras/Raf-1 signaling[22]. Our team did a series of studies on the analgesic effect of YXB on mice models with Complete Freund’s Adjuvant (CFA)-induced inflammatory pain in the previous period, and found that compared with IBU, YXB specialized in relieving chronic inflammatory pain. It can obviously suppress the expression of 30 inflammatory factors in the periphery of CFA mice, synergistically regulate the number, migration and function of macrophages, endothelial cells and microglia, and inhibit peripheral sensitization and central sensitization [23, 24]. In a word, our previous research has preliminarily elucidated the mechanism of YXB’s action on chronic inflammatory pain from the perspectives of anti-inflammatory and central sensitization. However, it is unclear whether the analgesic effect of YXB is related to inflammation resolution, and whether the pathways and mechanisms of action involved are related to the regulation of oxylipins.

This study mainly explores the characteristics and pathways of YXB’s role in promoting inflammation resolution and pain relief in CFA mice by regulating peripheral oxylipins. Oxylipins omics were applied to evaluate the influence of YXB on peripheral oxidized lipids in CFA mice, from which we discovered that YXB could regulate LXA4 characteristically. Furthermore, we verified the role of YXB in promoting inflammation resolution and alleviating pain through the LXA4 pathway. It was found that YXB can increase the expression of LXA4 in the paws, activate the formyl peptide receptor 2 (ALX/FPR2), and inhibit the expression of TRPA1. After administration of FPR2 antagonists, the number of CD68^+^cells and TRPA1 expression increased, suggesting that the pro-resolution and analgesic effects of YXB were hindered. In evaluating the effect of YXB on the pain model induced by the TRPA1 agonist AITC, it was suggested that YXB had limited effect on AITC- induced pain and did not significantly suppress TRPA1 expression, indicating that TRPA1 was a downstream signaling molecule regulated by YXB. Therefore, our study confirms that YXB can alleviate inflammation and pain in CFA mice by regulating the LXA4-FPR2-TRPA1 pathway.

## Materials and methods

### Drugs

**Fig. 1:**
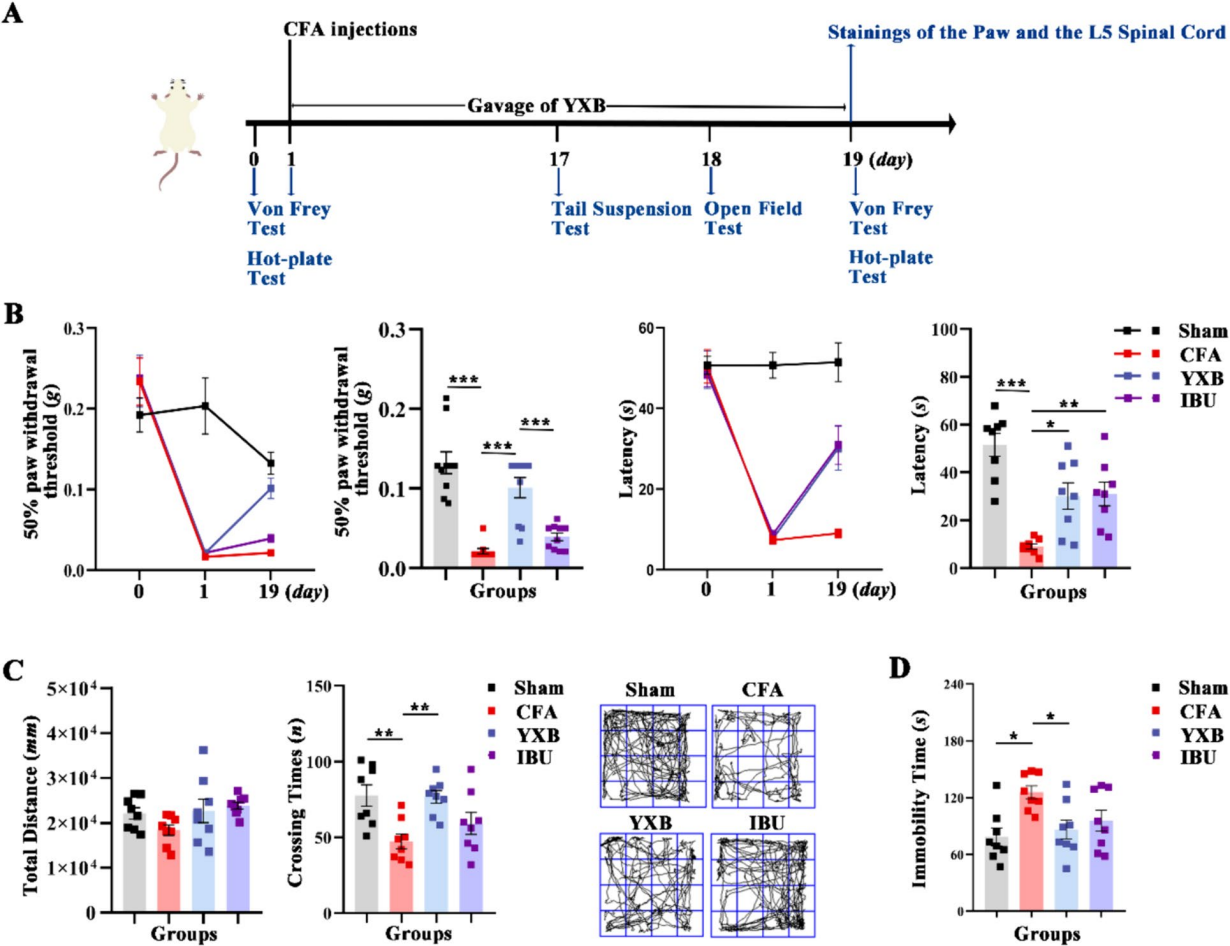
Effects of YXB on mechanical allodynia, heat hypersensitivity and depressive and anxious behavior in the CFA mice. **A** Schematic diagram of experimental design in this section. **B** The mechanical allodynia and heat hypersensitivity. **C** The Open Field Test (OFT) to evaluate anxious behavior. **D** The Tail Suspension Test (TST) to evaluate depressive behavior. All data are presented as (Mean ± S.E.M), *P < 0.05, **P < 0.01, ***P < 0.001

YXB was provided by Liaoning Huarun Benxi Sanyu Co., Ltd (batch no.20210117). The preparation process rigorously followed the standards set by the National Food and Drug Administration of China. Cyathula offfcinalis Kuan, Salvia miltiorrhiza Bunge and Astragalus membranaceus (Fisch.) Bunge was finely ground and sifted through a 100-mesh screen for subsequent use. A mixture including Salvia miltiorrhiza Bunge and Astragalus membranaceus (Fisch.) Bunge, among eight ingredients total, was decocted twice. The first decoction involved adding water at eight times the volume and boiling for 2 h; the second decoction involved adding water at six times the volume and boils for 1 h. The decoctions were then combined, filtered, and allowed to settle. The clear supernatant was vacuum-concentrated to a relative density of 1.18–1.22 at 50 °C to produce a clear paste, which was set aside. This paste was then granulated with the herbal powder, formed into granules, and mixed with sodium carboxymethyl starch and magnesium stearate. The mixture was then compressed into tablets, producing 1000 tablets, each coated with a thin film and containing 0.138 g per pill. The ingredient in the YXB showed by Supplementary Table 1. The main chemical components in YXB were obtained by applying high performance liquid chromatography-mass spectrometry (HPLC–MS) in the preliminary stage (Supplementary Table 2, Fig. 1) [22, 23]. According to the medication instructions, adults are advised to take 15 tablets daily, totaling 2.07 g. Drawing from a dosage conversion formula between mice and humans and previous empirical data, the effective doses of YXB were established at 0.2, 0.4 and 0.8 times the standard dose, equivalent to 60 mg/d/kg, 120 mg/d/kg, and 240 mg/d/kg, which were low, medium and high dosage respectively. Previous researches have verified that the high dosage yielded the most favorable outcomes [23, 24]. IBU was purchased from Huizhou Daya Pharmaceutical Co. (batch no.201802). The administered dose was 91 mg/kg, which corresponded to the clinically equivalent dose. Both YXB and IBU were dissolved in 0.5% carboxymethylcellulose sodium (CMC-Na) solution and administered by gavage every 12 h for 19 days.

### Experimental animals

8-week-old male SPF grade mice from Institute of Cancer Research (ICR), weighing 30 ± 2 g, were purchased from Spearfish (Beijing) Laboratory Animal Technology Co. [certificate no. SCXK (Jing) 2019-0010]. The experimental environment was the Animal Experiment Center of Institute of Basic Theory for Chinese Medicine, China Academy of Chinese Medical Sciences [license No. SYXK (Jing) 2021-0017]. The breeding environment was maintained at a temperature of 20 °C–24 °C, humidity of 40–60%. The mice were fed in separated cages, 5 mice per cage with free access to water and food. The operations involved in this experiment were in accordance with the requirements and standards of the Ethics Committee of the Institute of Chinese Materia Medica, China Academy of Chinese Medical Sciences.

### CFA animal model

As described [25], complete Freund’s adjuvant (CFA, 20 μL, Sigma-Aldrich, F5881) was injected subcutaneously into the left hind paws of mice. Sham group were injected with equal volume of saline.

### Experimental design and grouping

According to the objectives, the following design and grouping were carried out in our study: (1) To verify whether the analgesic effect of YXB in CFA mice is associated with the modulation of oxylipin-mediated inflammation resolution, the mice were randomly split into 4 groups: Sham, CFA, YXB-H (optimal dosage) [23, 24] and IBU.

(2) To verify the effect of YXB on the expression of LXA4, FPR2, TRPA1, Leukotriene A4 hydroxylase (LTA4H) in the paws of CFA mice, they were randomly divided into six groups: Sham, CFA, IBU, YXB-L, YXB-M and YXB-H.

(3) To further demonstrate whether the analgesic effect of YXB on CFA mice is related to the “LXA4-FPR2-TRPA1” pathway, two batches of animal experiments were performed. On the one hand, to assess whether the analgesic effect of YXB can be impaired after injection of FPR2 antagonist, the mice were randomly divided into four groups: Sham, CFA, YXB (YXB-H) and YXB-WRW4 (FPR2 antagonist, Selleck, S9818). In YXB-WRW4 group: for the CFA model, YXB was administered orally for 19 days, and on days 17–19, 45 min after the administration of YXB, WRW4(0.5 μg/μL) [26] was injected into the left hind paws of the mice. On the other hand, to evaluate whether TRPA1 is downstream of the YXB regulatory, the mice were randomly divided into three groups: Sham, AITC (TRPA1 agonist, Sigma-Aldrich, W203408) and YXB-AITC. Based on literature [27] and experimental experience, AITC (50 μM, 10 μL) was injected into the left hind paws of mice to construct a model. While for YXB-AITC group, AITC was injected into the left hind paws of mice 19 days after the pre-administration of YXB.

### Von Frey test

Mechanical allodynia thresholds were assessed by the Von Frey test. Briefly, the mice were acclimatized in the test frame for 30 min. When in the resting state, the mice were vertically stimulated with 0.008–0.4 g of Von Frey fiber wire at the plantar area of the left hind paws for 6–7 s. The presence of paw contraction, shaking or licking was recorded as positive. The operation was performed six times cumulatively, with an interval of 5 min. The 50% paw withdrawal threshold (PWT) was analyzed by up-down method [28].

### Hot plate test

Heat hypersensitivity was evaluated by the hot plate method. The test was performed before and after modeling and 1 h after drug administration on day 19. The mice were placed on the hot plate that was preheated to 53 °C, and the latency time was recorded when the mice showed reactions of foot lifting or licking [29].

### Tail suspension test

The depressive behavior of mice was assessed by Tail-suspension test (TST). As previously described [30], the mice were wrapped with tape around the tail tip at 1.5 cm and hung upside down on a tail suspension box for 6 min. The first 2 min served as the adaptation stage, and the accumulated time for the mouse to stop struggling was recorded in the last 4 min.

### Open Field test

The anxiety behavior of mice was evaluated by Open Field Test (OFT). Mice were placed in black cubic open-top box for free movement, the trajectories of mice were tracked and data were collected within 5 min by video analysis software (Shanghai Jiliang Software Technology Co., Ltd.). At the end of each batch of experiments, the field was wiped with ethanol to prevent the remaining odor from affecting the rest of the mice [31].

### Quantitative analysis of oxylipins

Left paws from five or six mice per group were chosen for oxylipins. According to the previous literature [32], supernatant was extracted from the samples after homogenization, centrifugation, re-extraction, elution, concentration, and re-dissolution. Liquid chromatography tandem-mass spectrometry (LC–MS / MS) platform (SCIEX Corporation, USA) and multiple reaction monitoring (MRM) mode were employed to perform qualitative and quantitative analyses of oxylipins in extracted samples.

In terms of chromatographic separation, the Waters ACQUITY UPLC HSS T3 C18 column (2.6 μm, 2.1 mm × 100 mm i.d.) was employed. The separation utilized a mobile phase consisting of two components: Phase A was a mixture of acetonitrile and water (60/40, v/v) with 0.04% acetic acid (Sigma-Aldrich, Germany), and Phase B comprised equal volumes of acetonitrile (Merck) and isopropanol (Merck). The column temperature was kept constant at 40 °C, with a flow rate of 0.4 mL/min and an injection volume of 10 μL. The gradient elution commenced with a 99.9:0.1 (v/v) ratio of A to B, adjusting to 70:30 (v/v) at 2 min, 50:50 (v/v) at 4 min, and moving to a 1:99 (v/v) ratio between 5.5 and 7 min, before returning to the initial ratio at 7.1 min. For mass spectrometry analysis, the electrospray ionization source temperature was adjusted to 550 °C, with the mass spectrometric voltage set to 5500 V in positive ion mode and − 4500 V in negative ion mode. The curtain gas pressure was maintained at 35 psi. Within the Q-Trap 6500 + system, ion pairs were scrutinized using optimized declustering potential (DP) and collision energy (CE) settings, ensuring precise identification and quantification of analytes.

Analyst^®^ 1.6.3 was used for processing mass spectrometric data. Mass spectrometric qualitative analysis of oxylipins relied on information from the oxylipin database. Differential metabolites were screened based on variable importance in projection (VIP > 1) using the orthogonal partial least squares discriminant analysis (OPLS-DA) model and independent samples t-test (P < 0.05). Subsequently, differential genes underwent heatmap analyses.

### Enzyme linked immunosorbent assay (ELISA)

Protein was extracted from the left paws of mice from each group, and the levels of LXA4 were measured according to the instructions of the ELISA kit (ELK Biotechology, China).

### Immunofluorescence

Perfused with 4% paraformaldehyde, the left paws, L4 ~ L5 segments of the DRG and spinal cords were taken and placed in fixative (4% paraformaldehyde + 8% sucrose solution) for 24 h. After 48 h of gradient dehydration in 15% and 30% sucrose solutions, respectively, the tissues were blotted dry with filter paper and collected. Coronal frozen sections with the thickness of 18 μm were prepared after embedding the tissue with OCT. Subsequently, immunofluorescence staining was performed. Sections were eluted three times with phosphate buffered saline (PBS). After blocking with 5% BSAT (5% Bovine albumin + 0.5% Triton X-100) for 30 min, the primary antibodies, namely, rabbit anti-LAT4H (Immunoway YT6624 1:400), rabbit anti-TRPA1 (Immunoway YN3017 1:400), rabbit anti-TRPV1 (abcam ab305299 1:500) mouse anti-IBA1(Invitrogen GT10312 1:400), mouse anti-CD68 (Immunoway YM3050 1:200) and mouse anti-CGRP (1:400) were incubated, and rested overnight at 4 °C. Sections were eluted three times with PBST and incubated fluorescent secondary antibodies: dylight goat anti-rabbit 488 (Earthox E032220 1:400) and dylight goat anti-mouse 594 (Earthox E032210 1:400) away from light for 1 h at ambient temperature. Subsequently, the nuclei were stained by DAPI (Solarbio C0065 1:1000). Finally, sections were dripped with antifade mounting medium and sealed. Fluorescence images were captured using laser confocal microscope (Olympus FV1000, × 600) and analyzed by ImageJ software.

### Flow cytometry

In line with literature [33] and our research group’s experience, fresh left paws of mice were processed to prepare a single-cell suspension through enzymatic digestion. Cells were aliquoted into flow cytometry tubes at a concentration of 1 × 10^6^ cells per tube, each supplemented with PBS up to 1 mL. The tubes were then centrifuged at 350 g for 5 min, the supernatant discarded, and the cells resuspended in 100 μL of stain buffer FBS 2 μL of Fc block was added, followed by a 5-min incubation at 4 °C. Antibodies CD45, CD11b, and F4/80 (BD Bioscience 463891, 557397, 566787) were then added, and the mixture was incubated for another 30 min at 4 °C. After adding 1 mL of PBS and another round of centrifugation at 350 g for 5 min, the supernatant was discarded, and the cells were resuspended in 500 μL of stain buffer FBS 2.5 μL of 7-AAD (BD Bioscience 559925) was added, and the mixture was incubated in the dark for 10 min, then filtered through a 70 μm mesh, and analyzed using a flow cytometer (BD Celesta). Statistical analysis that proportion of F4/80^+^CD11b^+^ cells among live cells in the paws was performed using FlowJo software.

### Western blotting

Protein was extracted from the left paws of mice, and its concentration was determined using the BCA method. After adding the loading buffer, the samples were boiled for 10 min to denature the proteins. Subsequently, the samples underwent 90-min electrophoresis with 80 V and 2-h wet transfer with 250 mA, followed by a 2-h blocking phase using 5% non-fat milk. Primary antibodies TRPA1 (1:1000) and β-actin (1:5000) were introduced and the mixture incubated overnight at 4 °C. The membranes were washed three times with 1% TBST, each wash lasting 15 min, and then incubated with secondary antibodies (1:5000) at room temperature for 2 h. Following three additional 15-min washes, the membranes were developed using an ECL detection reagent. β-actin served as the internal control. Protein bands were analyzed with Image J software to determine the relative expression levels of the TRPA1 protein.

### Statistical analysis

GraphPad Prism 8.0.2 was used for data analysis. Values are presented as mean ± standard error of the mean (x̄ ± S.E.M). Multigroup comparisons were made using analysis of variance. P < 0.05 indicated statistical significance.

## Results

### YXB alleviates mechanical allodynia and heat hypersensitivity as well as depressive and anxious behaviors in CFA mice

Studies have suggested that negative emotions are one of the characteristics of persistent inflammatory pain, and more than 85% of patients with chronic pain exhibit negative emotions such as depression and anxiety [34]. In our study, we not only evaluated the analgesic effect of YXB, but also focused on the effect of YXB on depressive and anxious behaviors in CFA mice (Fig. 1A).

In terms of analgesia, we compared PWT and thermal paw withdrawal latencies (PWL) in mice in each group before modeling, after modeling, and on the 19th day of administration. The results showed that there was no significant difference in PWT and PWL between the groups of mice before modeling, and on day 1 after modeling, PWT and PWL were significantly lower in the rest groups mice compared with the sham group. On the 19th day of administration, compared with the sham group, mice in the CFA group had significantly reduced in PWT and PWL. In contrast, compared with the CFA group, mice in the YXB group had significantly increased in PWT and PWL, and the IBU group had significantly increased in PWL (Fig. 1B). In terms of effects on emotional disorders, the total walking distance of the mice within 5 min and the crossing time in the central area were used to evaluate anxiety behavior, and a shorter total distance and crossing time indicated anxiety in mice. In the TST, depressive behavior was evaluated by the time the mice stopped struggling, and a longer time indicated that the mice were depressed. The results showed that compared with the sham group, mice in the CFA group had significantly shorter crossing time and longer stop- struggling time. Compared with the CFA group, mice in the YXB group had a significant increase in crossing time (Fig. 1C) and a significant decrease in time to stop struggling (Fig. 1D). Above indicated that YXB could alleviate mechanical allodynia and heat hypersensitivity as well as depression and anxiety behaviors in CFA mice.

### YXB promotes peripheral inflammation resolution and inhibits peripheral sensitization in CFA mice

**Fig. 2:**
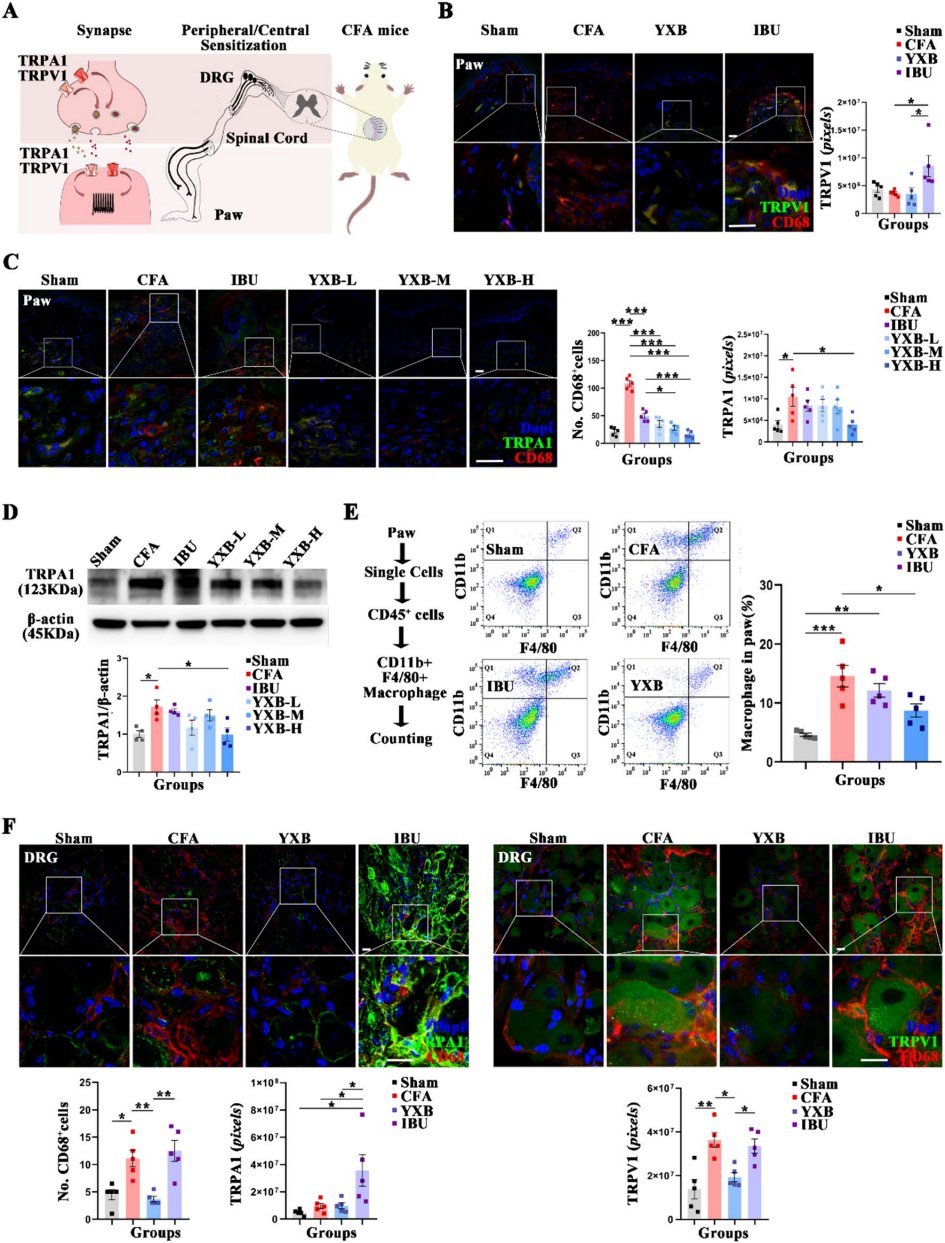
Effects of YXB on peripheral sensitization and resolution of peripheral inflammation in the CFA mice. **A** Expression of TRPA1 and TRPV1 on nociceptor (paws) and pain-transmitting primary neurons (DRG) is closely associated with inflammatory pain. **B** Expression of TRPV1 and CD68^+^ in the paws assessed by immunofluorescence (scale bar 50 μm) and statistical analysis with bar charts. **C** Expression of TRPA1 and CD68^+^ in the paws assessed by immunofluorescence (scale bar 50 μm) and statistical analysis with bar charts. **D** Expression of TRPA1 in the paws by western blotting and statistical analysis with bar charts. **E** F4/80^+^CD11b^+^ macrophage cells among live cells in the paws assessed by flow cytometry and statistical analysis with bar charts. **F** Expression of TRPA1, TRPV1 and CD68^+^ in DRG assessed by immunofluorescence (scale bar 50 μm) and statistical analysis with bar charts. All data are presented as (Mean ± S.E.M), *P < 0.05, **P < 0.01, ***P < 0.001

Appropriate clearance of inflammatory cells is the main characterization of inflammation resolution [35]. Macrophages are the main inflammatory cells involved in inflammatory pain [36], and CD68, F4/80^+^CD11b^+^ are marker for macrophages [37]. In the present study, the expression of CD68 in the paws and dorsal root ganglion (DRG) was assessed by immunofluorescence, the expression of F4/80^+^CD11b^+^ in the paws was measured by flow cytometry. The results showed that compared with sham group, the number of CD68^+^ and the proportion of F4/80^+^CD11b^+^ cells in the paws and DRG of mice in the CFA group was significantly increased (Fig. 2B, C, E and F); compared with the CFA group, the number of CD68^+^ and the proportion of F4/80^+^CD11b^+^ cells in the paws in the YXB group was significantly reduced (Fig. 2B, C and E), CD68^+^ in the DRG of mice in the YXB group was significantly reduced (Fig. 2F). It is suggested that YXB reduces the infiltration of peripheral macrophages and promotes the resolution of inflammation in CFA mice.

Peripheral sensitization is the main pathomechanism of inflammatory pain [38]. TRPs is a non-selective cation channel involved in peripheral sensitization, and the release of inflammatory mediators after tissue damage activates TRP channels, resulting in peripheral sensitization and inducing pain [39]. In the present study, the expression of TRPA1 and TRPV1 in peripheral injury receptors (paw) as well as in primary injury-sensing neurons (DRG) was measured by immunofluorescence. The results showed that compared with the sham group, TRPA1 expression in the paws significantly increased in the CFA group, while there was no significant difference in TRPV1 expression. Compared with the CFA group, TRPA1 expression significantly decreased in mice in the YXB-H group, while there was no significant difference in TRPV1 expression (Fig. 2B–D). Compared with the sham group, TRPV1 expression in DRG significantly increased in the CFA group, while there was no significant difference in TRPA1 expression. Compared with the CFA group, TRPV1 expression significantly decreased in mice in the YXB group, while there was no significant difference in TRPA1 expression (Fig. 2B and C). It is indicated that YXB inhibits peripheral sensitization by decreasing TRPV1 expression in DRG and TRPA1 expression in the paws.

### YXB modulate certain oxylipins in CFA mice and enhance the levels of LXA4

**Fig. 3:**
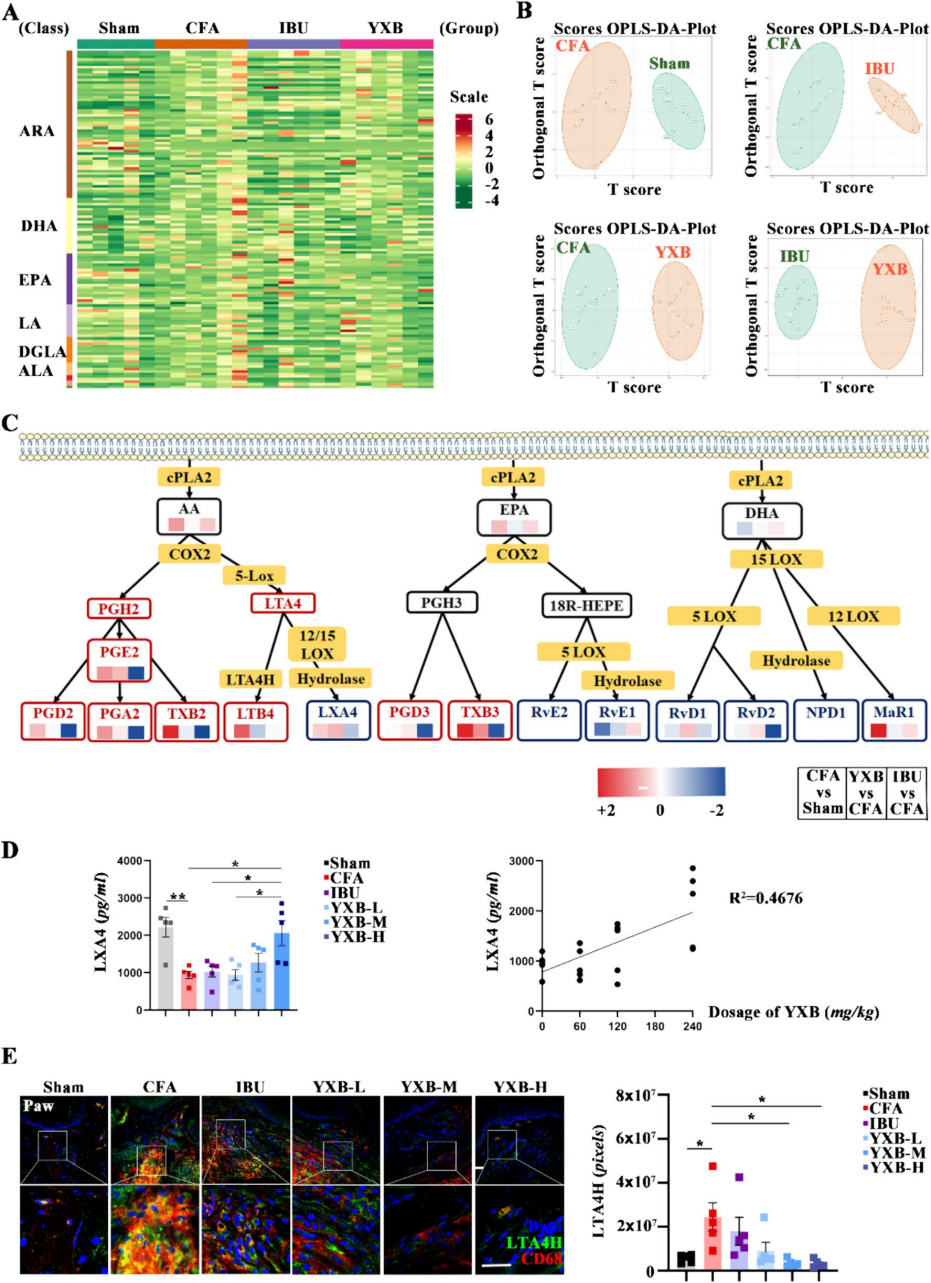
Effects of YXB on oxylipins in the CFA mice. **A** Heat map of oxylipins in the all samples of CFA mice. **B** Four pairs of Orthogonal Partial Least Squares-Discriminant Analysis (OPLS-DA) between the two groups (CFA vs Sham, YXB vs CFA, IBU vs CFA, YXB vs IBU). **C** Schematic diagram about YXB regulation of oxylipins in the CFA mice paws, the three small colored square boxes below each oxylipin represent the ratio of its expression between in CFA and Sham, YXB and CFA, IBU and YXB, respectively. The ratio is expressed as a logarithmic function with base 2. The red series represents a ratio greater than 1, meaning that the expression of the numerator is higher than the expression of the denominator, while the blue series represents a ratio less than 1, meaning that the expression of the numerator is lower than the expression of the denominator. **D** Expression of LXA4 in paws by ELISA and correlation analysis of LXA4 expression and YXB dose. **E** Expression of LTA4H in paws by immunofluorescence (scale bar 50 μm) and statistical analysis with bar charts. All data are presented as (Mean ± S.E.M), *P < 0.05, **P < 0.01

Considering the pivotal role of oxylipins in facilitating the resolution of inflammation and pain, we delved into the influence of YXB on peripheral inflammation resolution and sensitization. Subsequently, we explored YXB’s impact on peripheral oxylipins by conducting a quantitative oxylipin analysis in the paws of CFA mice (Fig. 3A and B). Out of 140 oxylipin varieties examined, 15 showed statistical significance. We visually depicted these 15 oxylipins, highlighting their sources, classifications, and expression changes. Diverging from ibuprofen’s mechanism, which predominantly suppresses the expression of prostaglandins and thromboxanes, YXB was found to elevate the levels of LXA4, RVD1, RVD2, and DHA in CFA mice, all known as SPMs (Fig. 3C). Quantitative oxylipins results, further validated by ELISA, indicated that compared with the sham group, there was a significant reduction in LXA4 expression in the oxylipins of CFA mice. Conversely, LXA4 expression significantly increased in the YXB-H treated CFA mice. These findings suggested that YXB can regulate the levels of certain DHA-category oxidized lipids in CFA mice’s paws and boost LXA4 expression (Fig. 3D). Furthermore, considering the negative correlation between LTA4H and LXA4 production, immunofluorescence staining was performed on the expression of LTA4H in the paws. The results showed that compared with the sham group, the expression of LTA4H in the paws of CFA group mice was significantly increased. Compared with the CFA group, the expression of LTA4H in the paws of YXB low, medium, and high dose groups mice significantly declined (Fig. 3E), indicating that YXB can inhibit the expression of LTA4H and thereby increase the content of LXA4.

### FPR2 antagonist can diminish the analgesic and pro-resolve effects of YXB

**Fig. 4:**
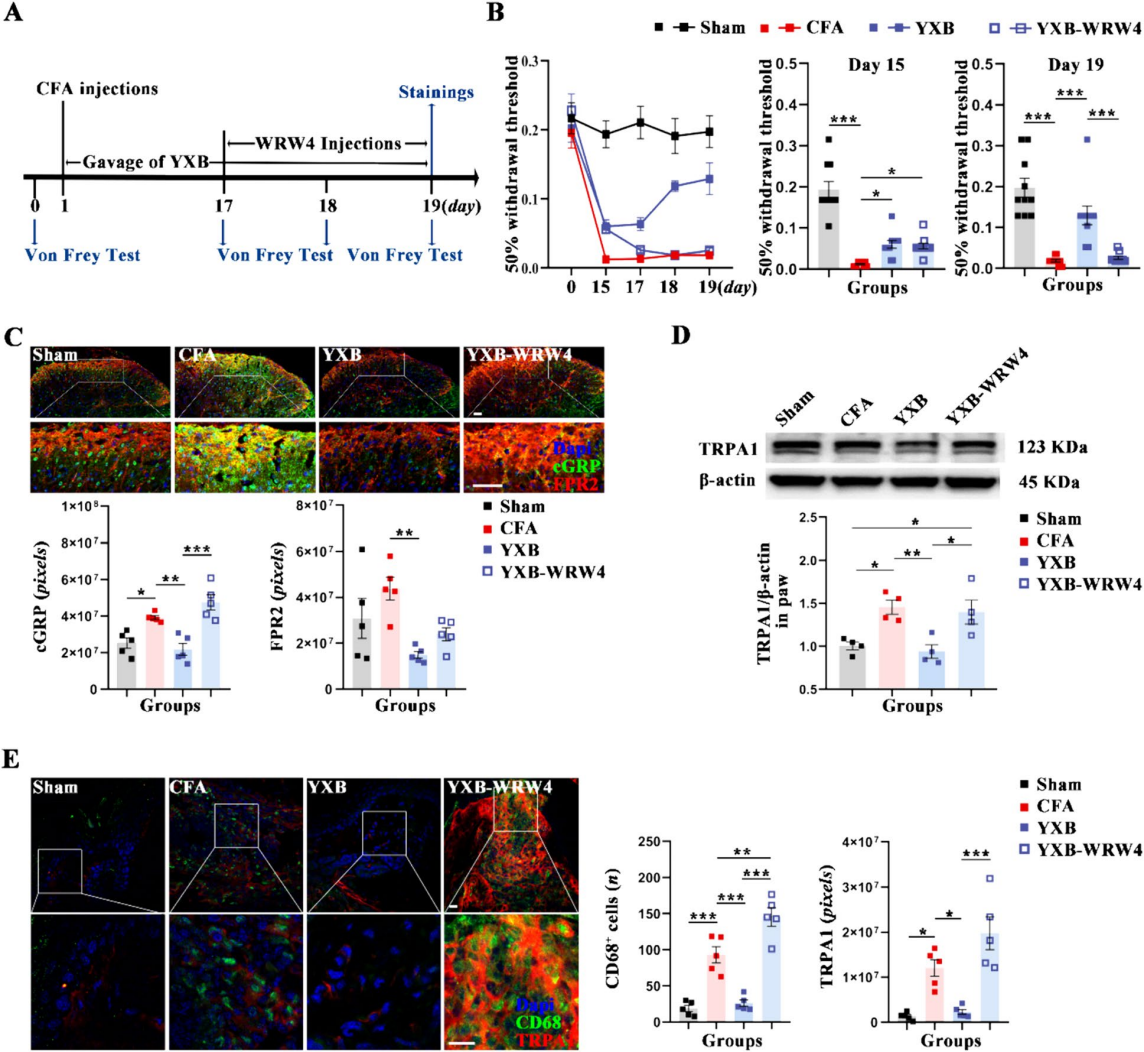
Effect of FPR2 antagonists on the analgesic effect of YXB in the CFA mice. **A** Schematic diagram of experimental design in this section. **B** Line and bar charts analysis of the mechanical allodynia. **C** Expression of CGRP and FPR2 in L5 spinal dorsal horn assessed by immunofluorescence (scale bar 50 μm) and bar charts analysis. **D** Expression of TRPA1 in the paws assessed by western blotting and bar charts analysis. **E** Expression of TRPA1 and CD68^+^in the paws assessed by immunofluorescence (scale bar 50 μm) and bar charts analysis. All data are presented as (Mean ± S.E.M), *P < 0.05, **P < 0.01, ***P < 0.001

Researches have indicated that activation of receptors by pro-resolution mediators can suppress TRPA1 or TRPV1 expression, which contributes to pain alleviation [19]. LXA4 primarily targets the FPR2 to reduce TRPA1 expression [19]. Our study reveals that YXB increases LXA4 levels while decreasing TRPA1 expression. Yet, its effect on FPR2 levels is not statistically significant, which leads to the hypothesis that YXB might modulate FPR2 activity. In this context, the FPR2 antagonist WRW4 was administered during days 17–19 of the YXB treatment in CFA mice to assess its effect on TRPA1 expression and YXB’s analgesic and pro-resolution efficacy (Fig. 4A). The analgesic effect of YXB was assessed by the measurement of PWT and the expression of Calcitonin Gene-Related Peptide (CGRP) in the L5 spinal dorsal horn. Additionally, the expression of CD68 in the paws served as an indicator for the inflammation-resolving capability of YXB. The results showed that compared with CFA group, on day 19, mice treated with YXB displayed a substantial increase in PWT (Fig. 4B), a significant decrease in CGRP expression in the L5 spinal dorsal horn (Fig. 4C), and a significant reduction in both TRPA1 expression and CD68^+^ cells in the paws (Fig. 4D and E). In contrast, compared with YXB group, mice in the YXB-WRW4 group exhibited a significant decrease in PWT (Fig. 4B) and increase in CGRP expression in the L5 spinal dorsal horn (Fig. 4C), as well as in TRPA1 expression and the number of CD68 ^+^ cells in the paws (Fig. 4D and E). These results indicated that the FPR2 antagonist may inhibit YXB’s analgesic and pro-resolving effects, indirectly confirming YXB’s role in inflammation resolution and pain relief via the “LXA4-FPR2-TRPA1” pathway. Additionally, the analysis of FPR2 expression in the L5 spinal dorsal horn revealed trends contrary to those observed in the paws, further suggesting that the LXA4 pathway constitutes a peripheral mechanism through which YXB mediates inflammation resolution.

### Limited impact of YXB on AITC-induced pain

**Fig. 5:**
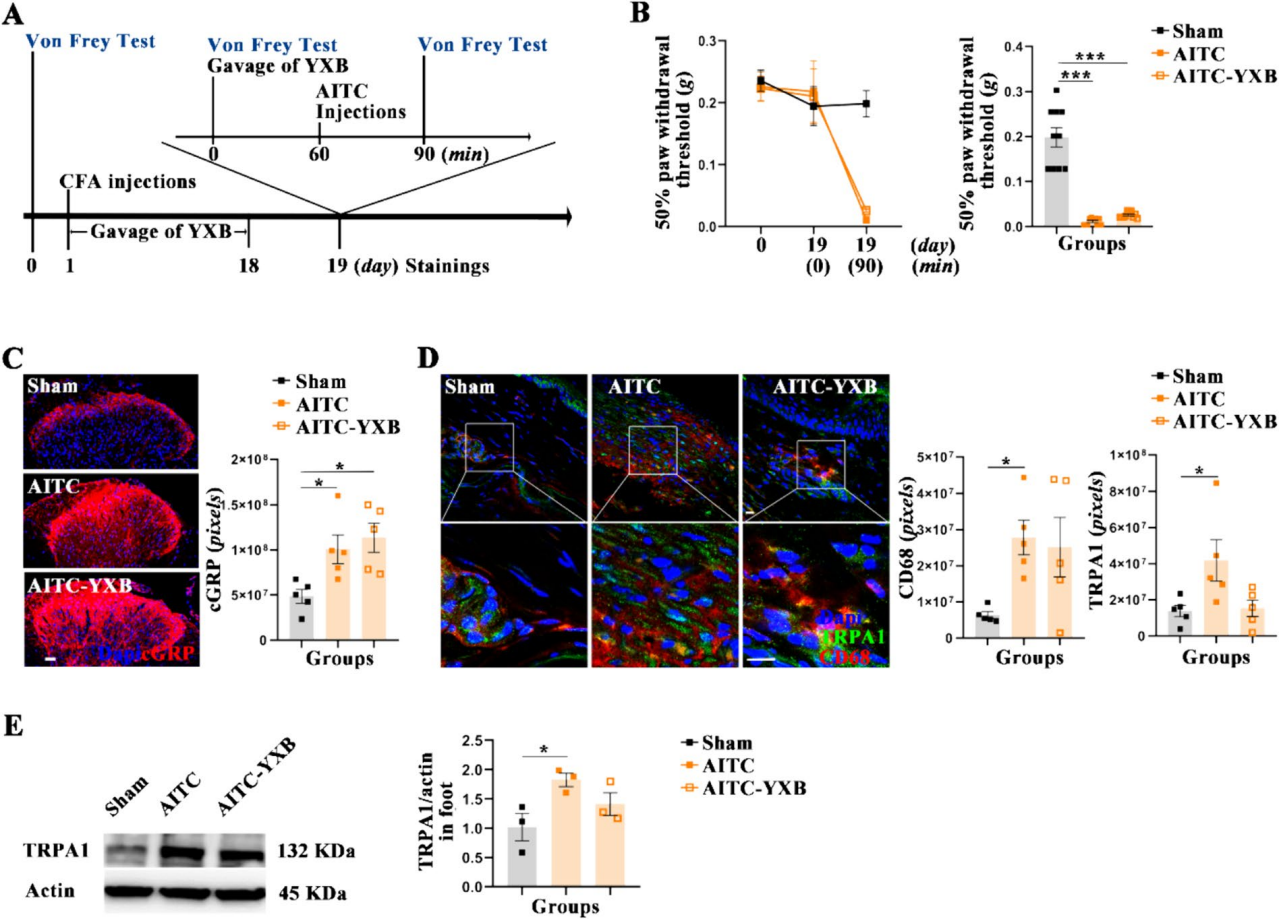
Limited effects of YXB on AITC-Induced pain. **A** Schematic diagram of experimental design in this section. **B** Line and bar charts analysis of the mechanical allodynia. **C** Expression of CGRP in L5 spinal dorsal horn assessed by immunofluorescence (scale bar 50 μm). **D** Expression of TRPA1 and CD68^+^ in the paws assessed by immunofluorescence (scale bar 50 μm) and bar charts analysis respectively. **E** Expression of TRPA1 in the paws assessed by western blotting and bar charts analysis. All data are presented as (Mean ± S.E.M), *P < 0.05, **P < 0.01, ***P < 0.001

In an effort to validate that YXB modulates TRPA1 via the LXA4 pathway, this study employed AITC, a known TRPA1 agonist, to induce pain and then evaluated YXB’s analgesic response. Given the transient nature of AITC-induced pain, a pre-treatment approach was adopted, as suggested by literature. The results demonstrated that, compared with the Sham group, mice in the AITC group experienced a significant reduction in PWT (Fig. 5B) and an increase in CGRP expression in L5 spinal dorsal horn (Fig. 5C), as well as a significant increase in the expression of TRPA1 and CD68 in the paws (Fig. 5D and E). Conversely, compared with the AITC group, mice treated with YXB prior to AITC exposure showed no significant differences in PWT (Fig. 5B) or CGRP expression in the L5 spinal dorsal horn (Fig. 5C), nor in TRPA1 and CD68 expression in the paws (Fig. 5D and E). This suggests that YXB’s effect on the AITC-induced pain model is limited, indicating TRPA1 as a downstream target regulated by YXB.

## Discussion

Inflammatory pain, a prevalent chronic condition, is frequently associated with negative emotional states, including depression and anxiety [40]. The optimal treatment strategy for inflammatory pain would address not only the pain itself but also the accompanying emotional distress. NSAIDs are commonly prescribed as the initial treatment option for managing inflammatory pain. However, their efficacy in cases of persistent inflammatory pain remains limited, and long-term usage is not recommended due to potential adverse effects. Injection of CFA into the paws is a well-established and reliable method for generating animal models of inflammatory pain. Studies utilizing the CFA mice model have consistently demonstrated behaviors reflective of anxiety and depression [41]. YXB, a medication approved in Traditional Chinese medicine for the management of inflammatory conditions such as RA, has shown promise in clinical applications for its analgesic properties. However, there are currently few reports on the pharmacological research of YXB. Our team mainly focused on two models, respectively RA rats and mice with CFA induced inflammatory pain. The research showed that YXB could reduce joint swelling, improve mechanical allodynia and heat hypersensitivity as well as inhibit pannus formation in RA rats by regulating LOX/Las/Raf-1 signaling [22]. In the study of the effect of YXB on CFA mice, initial findings indicated that YXB could elevate the mechanical allodynia thresholds in CFA mice in a manner that is both time-sensitive and dose-dependent [23]. Current study extends these observations by demonstrating that YXB not only enhances the mechanical pain threshold but also ameliorates heat hyperalgesia in CFA mice, which further enriches the evidence of YXB’s analgesic effect on CFA mice. Furthermore, assessments utilizing open field and tail suspension tests have corroborated the presence of anxiety and depression-like behaviors in CFA mice, which is a finding in line with previous researches. Notably, YXB treatment effectively mitigates these behaviors. Therefore, this study contributes to the pharmacological understanding of YXB, highlighting its therapeutic effects on inflammatory pain, and expanding the groundwork laid by prior research.

Inflammation is a natural immune response with self-limitation. When the stimulating factors of inflammation are managed, the damaged tissue tends to repair status and shift to the inflammation resolution phase. Blocked inflammation resolution carries the potential risk of persistent inflammation [42]. Meanwhile, it has been shown that early anti-inflammatory treatment in the initial stage of inflammation can affect the production of pro-inflammatory mediators, inhibit inflammation related cells, and thus inhibit inflammation resolution [43]. This is also one of the main reasons why inflammatory pain persists and NSAIDs such as IBU have limited effects in the later stages of inflammatory pain. Therefore, it is crucial for a wide range of chronic inflammatory diseases, including inflammatory pain, to carry out the pro-inflammatory resolution strategy. Inflammatory resolution involves the blockade of pro-inflammatory signaling, production of anti-inflammatory mediators, and proper clearance of inflammatory cells including macrophages [44]. Our preliminary study found that YXB had a cumulative effect on the intervention of CFA mice, with a significant effect during the middle and later stages of inflammatory pain, and could also inhibit the expression of peripheral pro-inflammatory factors. CD68^+^ and F4/80^+^CD11b^+^cells are recognized markers of mature macrophages that significantly increases in CFA induced inflammatory pain models [37, 45]. The present study has evaluated the effect of YXB on the resolution of peripheral inflammation in CFA mice by examining the expression of macrophages, namely the number of CD68 ^+ ^cells and F4/80^+^CD11b^+^cells, in the peripheral nociceptor swelling paws and the number of CD68^+^ cells of pain primary transmission neuron DRG. We have found that YXB can significantly reduce the number of CD68^+^ cells and and the proportion of F4/80^+^CD11b^+ ^cells in the paws of CFA mice, reduce macrophage infiltration, and facilitate inflammation resolution.

Inflammation resolution is actively monitored by endogenous pro-resolving mediators, notably oxylipins, which has shown efficacy in treating conditions such as inflammatory pain [46], atherosclerosis [47], tumors [48], and RA [49] without significant adverse effects. Consequently, targeting these pro-resolving mediators to promote inflammation resolution and alleviate pain emerges as a critical strategy. Researches have indicated that the patterns and abundance of oxylipins expression, pivotal in inflammation regulation, are consistent across humans and rodents [50]. The current study explored the impact of YXB on the regulation of oxylipins within the paws of CFA mice. Employing oxylipins omics, the study revealed that diverging from IBU’s mechanism, which predominantly suppresses the expression of pro-inflammatory agents such as prostaglandins and thromboxanes, YXB modulated the levels of pro-resolving mediators like LXA4, RVD1, and RVD2, with a marked elevation in LXA4 levels. This finding elucidated the distinct therapeutic benefits of IBU and YXB in the management of inflammatory pain. IBU effectively counters inflammation in the acute phase through its anti-inflammatory action. In contrast, YXB exhibits a delayed response but facilitates inflammation resolution by modulating pro-resolving mediators, thereby offering analgesic effect during the middle and later stages of inflammatory pain.

Lipoxins are lipid mediators that originate from arachidonic acid (AA) and are produced through a transcellular process involving the catalysis of AA by various lipoxygenases (LOs) [51]. These compounds are pivotal in the modulation of numerous acute and chronic inflammatory conditions, cancer, pain, and cerebrovascular diseases, thanks to their anti-inflammatory and inflammation-resolving characteristics [52]. Specifically, LXA4 is notable for its ability to diminish inflammatory pain by elevating mechanical allodynia thresholds and suppressing heat hyperalgesia [16, 53]. Researches showed that LTA4H was closely related to the generation of LXA4. Inhibiting LTA4H could induce lipid mediator class switching in the 5-lipoxygenase pathway and improve LXA4 biosynthesis [54]. This study found that YXB could significantly inhibit the expression of LTA4H in the paws of CFA mice, indicating that YXB can increase LXA4 generation by inhibiting LTA4H expression. Besides, studies showed that mediators could promote resolution, mitigate pain and facilitate the resolution of inflammation by engaging their specific ligands and by reducing the activity or expression of TRPA1 and TRPV1 [19]. LXA4 mainly targets the G protein-coupled ALX/FPR2 receptor. Activating this receptor in the periphery could lessen pain hypersensitivity in mice subjected to CFA-induced inflammatory pain [55] and also curb TRPA1 activity [26, 56]. TRPA1, part of the TRP channel family, is acknowledged as a key therapeutic target for chronic pain management. Activating or boosting TRP channels in pain receptors (nociceptors) may increase neuronal firing, resulting in hyperalgesia [27]. Researches showed a significant increase in the expression of TRPA1 in the paws of mice model with inflammatory pain [57]. In our research, we evaluated the expression levels of LXA4, FPR2, and TRPA1 in the paws of mice treated with CFA and observed that YXB treatment notably enhanced LXA4 expression while suppressing TRPA1 expression, without markedly altering FPR2 levels. Administration of the FPR2 antagonist led to a significant rise in CD68 and TRPA1 expression in the paws. This suggests that YXB’s influence on FPR2 may be through its modulatory effects. Further experimental validation revealed that YXB had a modest analgesic impact on pain induced by AITC, indicating that TRPA1 acts as a downstream link regulated by YXB. In conclusion, our findings suggest that YXB alleviate inflammation and pain in CFA mice by modulating the LXA4-FPR2-TRPA1 pathway, and the regulation of LXA4 can be achieved by inhibiting the expression of LTA4H.

In summary, YXB can significantly regulate the pro-resolving mediators of paws in CFA mice to promote the resolution of inflammation. The LXA4-FPR2-TRPA1 signaling pathway is a key mechanism for the inflammation resolution and analgesic effects. This study reveals the mechanism that YXB promotes inflammation resolution based on oxylipins omics, providing experimental evidence for the clinical application of YXB in the treatment of chronic inflammatory pain and related diseases.

### Supplementary Information

Additional file 1.

## Data Availability

All data generated or analysed during this study are included in this published article.

## Abbreviations

CFA: Complete Freund’s adjuvant
CGRP: Calcitonin gene-related peptide
DRG: Dorsal root ganglia
FPR2: Formyl peptide receptor 2
IBU: Ibuprofen
LTA4H: Leukotriene A4 hydroxylase
LXA4: Lipoxins A4
NSAIDs: Non-steroidal anti-inflammatory drugs
OFT: Open field test
SPMs: Specialized pro-resolving mediators
TRPA1: Transient receptor potential ankyrin 1
TRPV1: Transient receptor potential vanilloid 1
TST: Tail-suspension test
