# Supplementary material for: The analgesic effects of Yu-Xue-Bi tablet (YXB) on mice with inflammatory pain by regulating LXA4-FPR2-TRPA1 pathway
Source: Chin Med. 2024 Aug 6;19:104. doi: 10.1186/s13020-024-00975-1 (PMC11302111; doi:10.1186/s13020-024-00975-1)
Supplement: Supplementary file 1 — Additional file 1. [file 13020_2024_975_MOESM1_ESM.pdf]

Table 1 The composition and dosage of YXB.

Plant name	Chinese name	English name	Crude drug (g) in daily prescription	Manufacturer batch number
Boswellia carteri Birdw	Ruxiang	Olibanum	1.1 g	180601
Commiphora myrrha (Nees) Engl	Moyao	Myrrha	1.1 g	180601
Clematis chinensis Osbeck	Weilingxian	Clematidis radix et rhizoma	2.7 g	181101
Cyathula offfcinalis Kuan	Chuanniuxi	Cyathulae radix	2.7 g	180401/181201
Curcuma longa L	Jianghuang	Curcumae longae rhizoma	1.8 g	180601
Carthamus tinctorius L	Honghua	Carthami ffos	1.8 g	181101
Salvia miltiorrhiza Bunge	Danshen	Salviae miltiorrhizam radix et rhizoma	3.6 g	181101
Cyperus rotundus L	Xiangfu	Cyperi rhizoma	2.2 g	181001
Ligusticum chuanxiong Hort	Chuanxiong	Chuanxiong rhizoma	2.7 g	181201
Astragalus membranaceus (Fisch.) Bunge	Huangqi	Astragali radix	2.7 g	181204
Angelica sinensis (Oliv.) Diels	Danggui	Angelicae sinensis radix	1.8 g	180602

Table 2 Identification of chemical constituents of YXB.

Chemical constituents	Formular	Negative ion chromatogram	Positive ion chromatogram	Retention time
Hydroxysafflor yellow A	$C_{27}H_{32}O_{16}$	611.1143	613.1873	10.480
Oleanolic acid	$C_{30}H_{48}O_3$	455.1431	457.1545	11.196
Calycosin glycoside	$C_{22}H_{22}O_{10}$	491.0821	447.1350	15.345
Salvianolic acid B	$C_{36}H_{30}O_{16}$	717.0913	719.1720	21.088
Cyasterone	$C_{29}H_{44}O_8$	519.0541	521.1151	21.095
Astragaloside IV	$C_{41}H_{68}O_{14}$	829.3964	-	27.594
Curcumin	$C_{21}H_{20}O_6$	-	369.1390	29.001

Cryptotanshinone	C19H20O3	-	297.1525	30.208
Tanshinone IIA	C19H18O3	-	277.1835	30.238
Tanshinone I	C18H12O3	-	295.1367	30.820

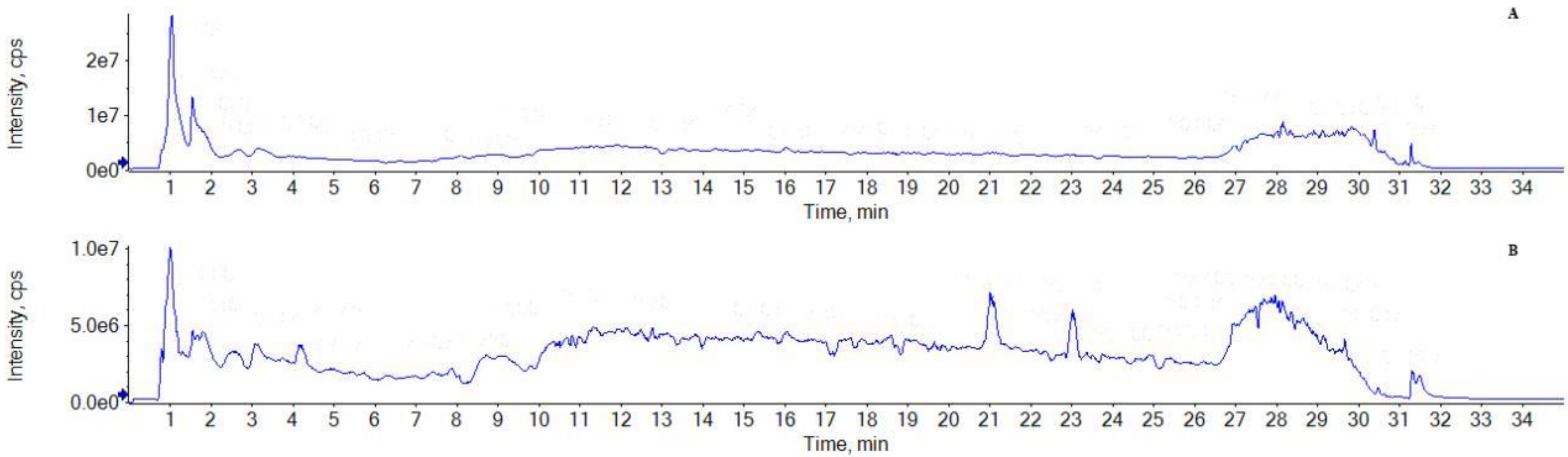

Fig. 1. Sample positive and negative ion chromatogram. (A) Sample positive ion chromatogram. (B) Sample negative ion chromatogram.

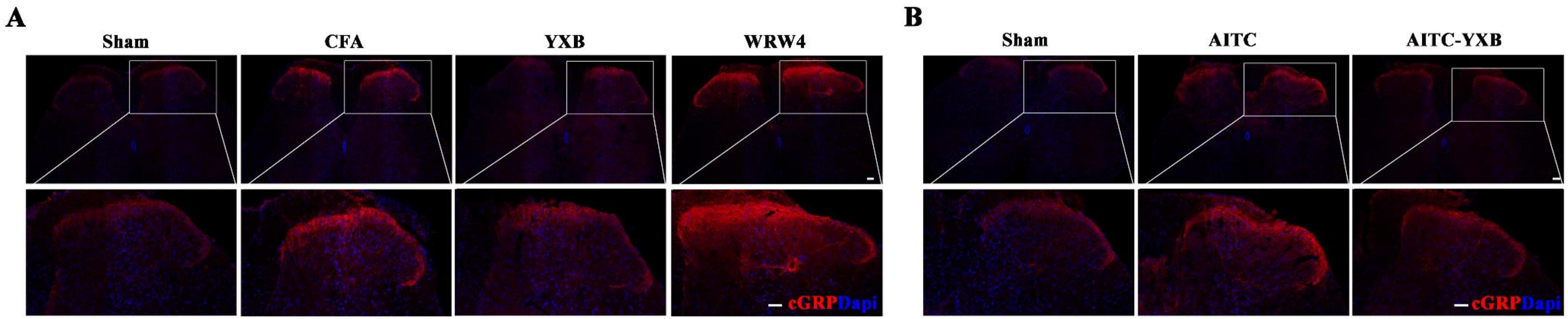

Fig. 2. Expression of CGRP in L5 spinal dorsal horn assessed by immunofluorescence. (scale bar 50 μm, X 100). (A) Expression of CGRP in L5 spinal dorsal horn among group of Sham, CFA, YXB and WRW4. (B) Expression of CGRP in L5 spinal dorsal horn among group of Sham, AITC and AITC-YXB.
